# Prognostic factors for long-term improvement in pain and disability among patients with persistent low back pain

**DOI:** 10.1186/s12998-024-00546-z

**Published:** 2024-06-25

**Authors:** Elin Mihlberg, Bodil Al-Mashhadi Arnbak

**Affiliations:** 1 Center of Muscle and Joint health, Department of Sports Science and Clinical Biomechanics, University of Southern Denmark, Campusvej 55, Odense M, DK-5230 Denmark; 2 Department of Radiology, Hospital Lillebaelt, Beriderbakken 4, Vejle, DK-7100 Denmark

**Keywords:** Disability, Low back pain, Prognosis, Prognostic factors, Cohort, Longitudinal, Secondary sector

## Abstract

**Background:**

Prognostic research in low back pain (LBP) is essential for understanding and managing the condition. This study aimed to, (1) describe the proportions with mild-moderate and severe pain and disability at baseline, 1-year and 4-year follow-up, and (2) investigate prognostic factors for improvement in pain and disability over 4 years in a cohort of secondary care LBP patients.

**Methods:**

This was a secondary analysis of a cohort of patients with LBP aged 18–40 years recruited from a non-surgical outpatient spine clinic between March 2011 and October 2013 (*n* = 1037). Questionnaires were collected at baseline, 1-year, and 4-year follow-up. Disability was assessed using the Roland Morris Disability Questionnaire (RMDQ 0-100) and pain intensity using the Numeric Rating Scale (NRS 0–10). ’Mild-moderate pain’ was defined as NRS < 7 and ’severe pain’ as NRS ≥ 7. Likewise, ’mild-moderate disability’ was defined as RMDQ < 58.3, and ’severe disability’ was RMDQ ≥ 58.3. In the prognostic analysis, improvement in pain and disability over 4 years was defined as meeting both criteria: decrease of ≥ 2 on the NRS and of ≥ 20.8 on the RMDQ. Sixteen candidate prognostic factors were assessed by multivariate logistic regression.

**Results:**

Among patients with information available at all three time points (*n* = 241), 54%/48% had persistent mild-moderate pain/disability, while only 7%/15% had persistent severe pain/disability. Of patients included in the multivariate prognostic analysis regarding improvement over 4 years (*n* = 498), 32% had improved in pain and disability after 4 years. Positive associations were found for pain intensity (OR 1.34 [95%CI: 1.17–1.54]), disability (OR 1.01 [1.00-1.02]), and regular employment or studying (OR 1.67 [1.06–2.64]), and negative associations for episode duration (OR 0.99 [0.99-1.00]) and risk of persistent pain (OR 0.58 [0.38–0.88]).

**Conclusion:**

Patients with persistent LBP in secondary care had mostly mild-moderate pain and disability consistently at all three time points, with few having consistently severe symptoms over 4 years. Moreover, approximately half of the included patients improved in pain and disability. We found that pain intensity, disability, episode duration, regular employment or studying, and risk of persistent pain predicted a long-term improvement. However, the limited availability of complete follow-up data may affect generalisability.

**Supplementary Information:**

The online version contains supplementary material available at 10.1186/s12998-024-00546-z.

## Background

Low back pain (LBP) is often a self-limiting condition, but a subset of individuals develop persistent symptoms. Persistent LBP is both disabling and detrimental to patients’ quality of life [1]. Patients with LBP typically experience recurrent episodes and fluctuations, and studies have shown that an early improvement is followed by little change after six weeks [2, 3]. Thus, the population-averaged course of LBP does not necessarily represent the individual, but patients follow different LBP trajectories [4, 5].

Prognostic research in LBP is essential to improve the understanding of the condition and its management [6, 7]. Firstly, knowledge of the course of LBP and prognostic factors will help patients to better understand their back pain. LBP is complex, and a better understanding can provide the patients with valuable information about what to expect, enabling them to make informed decisions about their treatment options, engage in self-management strategies, and set realistic expectations for their recovery. In addition, prognostic knowledge will enable clinicians to adapt and individualise management strategies, and a targeted approach to prognostic factors may optimise patient care.

Numerous studies have investigated the association between baseline prognostic factors and various outcomes related to LBP. Several baseline factors have been consistently found to predict LBP outcomes, such as age, gender, pain intensity, episode duration, previous episodes of LBP, leg pain, disability, work status, depression, fear-avoidance, and recovery expectations [8–18]. However, most previous studies used shorter follow-up periods of 12 months or less, and only a few used 4–5 years [8–10, 19]. The limited number of long-term studies may be problematic because prognostic factors may differ between the short and long term [8, 10, 11]. Consequently, our understanding of long-term prognostic factors for LBP remains limited.

Thus, this study aimed to investigate potential prognostic factors for improvements in pain and disability over 4 years in patients referred to a spine center with LBP. Specifically, we aimed to (1) describe the proportions with mild-moderate and severe pain and disability at baseline, 1-year and 4-year follow-up, and (2) investigate prognostic factors for improvement in pain and disability over 4 years in patients with LBP referred to a spine center.

## Methods

### Study design

This study was a secondary analysis of longitudinal data from the Spines of Southern Denmark (SSD) cohort [20].

### Study participants

Study participants were recruited from the Spine Center of Southern Denmark between March 2011 and October 2013. Patients were referred to the Spine Center by medical specialists in primary care, chiropractors, or other hospital departments, for a multidisciplinary assessment. The guidelines for referral to the Spine Center during the baseline study period were: (1) an episode of back pain lasting 2–12 months, and (2) insufficient effect to conservative treatment in primary care. Patients aged 18–40 years who were referred with LBP as their primary complaint were consecutively included in the study (*n* = 1037). Details of the inclusion and exclusion process have been reported previously [20].

As part of the standard procedure at the Spine Center follow-up questionnaires were sent out to all patients 1 year after their first visit. All cohort participants were also invited by letter to participate in the 4-year follow-up, which was conducted between November 2014 and June 2017 using the same questionnaires [21]. The questionnaire data were entered directly into the electronic SpineData database [22].

### Outcome measures of pain and disability

Pain was assessed using the Numeric Rating Scale (NRS) (range 0–10) [23]. Disability was assessed using the 23-item version of the Roland Morris Disability Questionnaire (RMDQ) [24], which includes information on daily physical activities and functions [24, 25]. The original score was converted to a proportional score (range 0-100) to better deal with missing values.

To describe the proportions with different symptom severity over the follow-up periods, ‘mild-moderate pain’ was defined as NRS < 7 and ‘severe pain’ was defined as NRS ≥ 7 [26], and ‘mild-moderate disability’ was defined as RMDQ < 14 and ‘severe disability’ was defined as RMDQ ≥ 14 [27]. The RMDQ score of 14 was converted to a corresponding proportional score of 58.3.

The proportion of participants with improvements in pain and/or disability, between baseline and 4-year follow-up were assessed according to previously defined minimal clinical changes. For the NRS, a cutpoint of ≥ 2 was used, and for the RMDQ, a cutpoint of ≥ 5, which was recalculated to a corresponding proportional score of 20.8 [28]. In the prognostic analysis improvement was defined as meeting both of these criteria. Participants were excluded from the prognostic analysis if they were unable to improve from their baseline score, i.e., participants with an NRS < 2 or RMDQ < 5 at baseline.

### Candidate prognostic factors

The variables available for selection were those collected as part of the standard procedure at the Spine Center included in the SpineData database. These variables were included based on evidence of their role in the diagnosis, prognosis, or treatment of spinal pain [22].The final selection of candidate prognostic factors to be included in the analysis was based on the updated literature and research group discussions with colleagues who have extensive experience in prognostic research in LBP. The recommendation of at least 10 events for each investigated prognostic association was applied [29], together with a maximum of 10% missing values.

### Statistical analyses

Data analyses were performed using STATA software, version 17.0.

Differences in baseline characteristics between participants included and excluded from the analysis were assessed using the Wilcoxon rank-sum test and Pearson’s chi-squared test, as appropriate.

The proportions of participants with mild-moderate and severe pain and disability, respectively, were used to create Sankey diagrams to visually depict the progression of symptoms between baseline, and 1-year, and 4-year follow-up, using SankeyMATIC.

Logistic regression models were used to examine the association between the candidate prognostic factors and improvement in pain and disability at the 4-year follow-up. First, univariate logistic regression was performed for each of the candidate prognostic factors and improvement in pain and disability. Candidate prognostic factors with a *p*-value ≤ 0.2 and a Spearman rank correlation coefficient that was low (+/- 0.5) [30, 31] were then entered into a backward stepwise multiple logistic regression (model 1).

A Hosmer-Lemeshow goodness-of-fit test was performed to test for model misfitting. Because of the known limitations of stepwise regression [32, 33], the performance of two additional models was assessed by area under the curve (AUC): a multivariate non-stepwise regression including all 16 variables (model 2) and an extended stepwise regression including all variables (model 3).

*P*-values ≤ 0.05 were considered significant for all analyses.

## Results

**Fig. 1:**
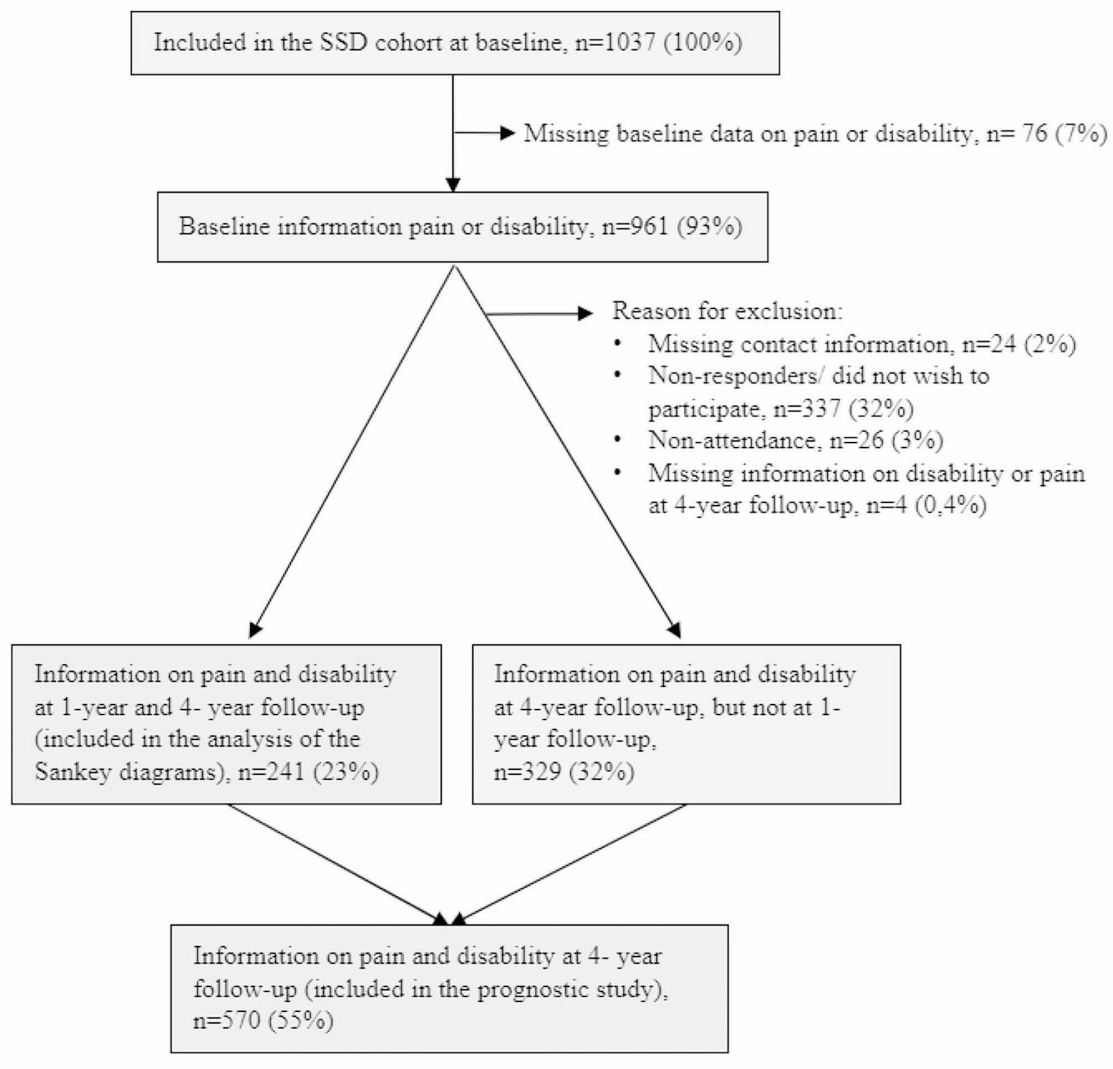
Flow chart of inclusion of patients from the Spines of Southern Denmark (SSD) cohort

Figure 1 shows the flow chart for inclusion in the study and the reasons for dropout.

Of the 1037 participants included at baseline, 241 (23%) had data available for both pain intensity and disability at baseline, 1-year, and 4-year follow-up and were included in the analysis of the Sankey diagrams. The median age of these 241 participants at baseline was 33.9 years, 123 (51%) were female, and the median duration of the episodes was 8.6 months. These patients were slightly older and were slightly more likely to report previous episodes of LBP, to be regularly employed or studying, and slightly less likely to report fear-avoidance than those not included. No other significant differences were found (Additional file 1).

Of the 1037 patients included at baseline, 570 (55%) had information on both pain and disability at baseline and 4-year follow-up and were included in the prognostic analysis. The median age of the included participants was 33.1 years, 308 (54%) were female, and the median episode duration was 10.1 months (Table 1). Participants included in this analysis were slightly older, and they were slightly more likely to report previous episodes of LBP and leg pain than those not included. No other significant differences were found (Table 1).

**Table 1:** Baseline characteristics of the included and the not-included patients in the 4-year follow-up study

Baseline variables ^a^	Included		Not included ^b^	
	Missing values, *n* (%)		Missing values, *n* (%)	
*Contextual factors*				
Age (years), median (IQR)	0 (0)	33.1 (27.5–37.4)*	0 (0)	31.8 (25.7–36.3)*
Sex (female)	0 (0)	54 (49.4–57.6)	0 (0)	54 (49.6–58.7)
Normal weight (BMI)	25 (4)	44 (40.0-48.4)	88 (18.8)	47 (41.9–52.0)
*Pain-related factors*				
Pain intensity (NRS), median (IQR)	0 (0)	6 (4.7–7.3)	59 (12.6)	6.3 (5.0-7.3)
Episode duration (months), median (IQR)	17 (3)	10.1 (3.7–35.6)	65 (13.9)	11.1 (3.8–44.8)
Previous LBP episodes	11 (1.9)	77 (73.8–80.7)*	68 (14.6)	70 (65.7–74.7)*
Leg pain	7 (1.2)	83 (80.0-86.2)*	66 (14.1)	77 (73.2–81.4)*
Pain in other parts	13 (2.3)	42 (37.7–45.9)	68 (14.6)	40 (34.8–44.4)
*Activity limitation restricted factors*				
Disability (RMDQ), median (IQR)	0 (0)	56.5 (39.1–73.9)	76 (16.3)	56.5 (39.1–73.9)
*Psychological factors*				
Depression	11 (1.9)	22 (18.2–25.1)	84 (18)	23 (18.7–27.2)
Anxiety	8 (1.4)	45 (40.7–48.6)	83 (17.8)	44.5 (39.5–49.5)
Fear-avoidance	7 (1.2)	21 (17.92–24.7)	75 (16.1)	26 (21.9–30.7)
Risk of persistent pain	8 (1.4)	46 (41.95–50.2)	83 (17.8)	45 (40.3–50.3)
Social isolation	8 (1.4)	12 (9.4–14.8)	83 (17.8)	14 (10.8–17.8)
*Participation restricted related factors*				
Regular employment or studying	14 (2.5)	71 (66.7–74.3)	74 (15.8)	33 (28.7–38.0)
Sick leave	46 (8)	50 (45.5–54.1)	97 (20.8)	51 (45.7–55.9)

Included participants; *n* = 570, Not-included participants; *n* = 467.

a: Unless otherwise stated, values are the percent of patients (95% confidence interval).

b: Participants included in the baseline cohort, but not in the analysis of the prognostic factors.

IQR: interquartile range, BMI: Body Mass Index, RMDQ: Roland Morris Disability Questionnaire (23-item version); NRS: Numerical Rating Scale.

*P-value < 0.05

### Course of pain and disability

**Fig. 2:**
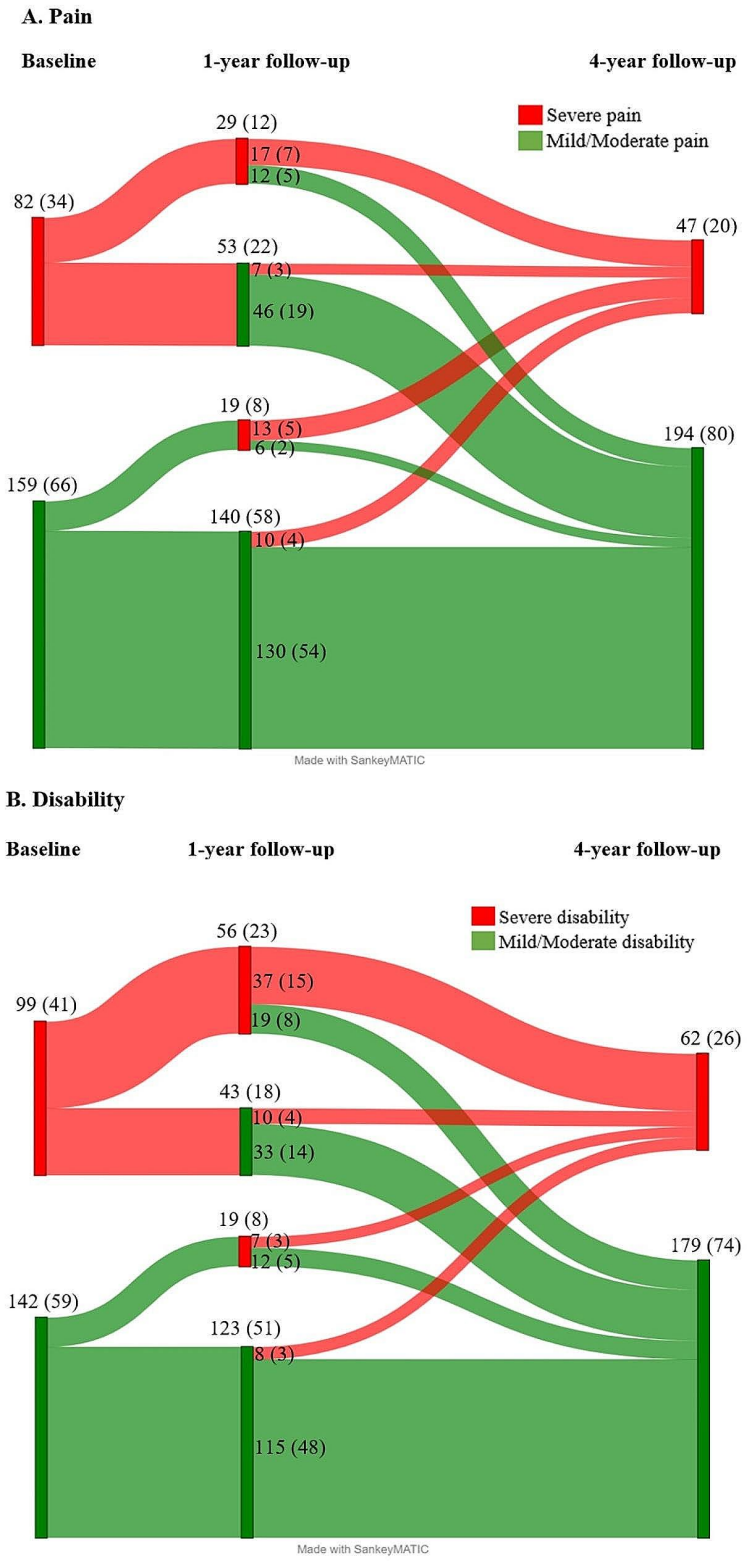
Sankey diagrams with the proportions with mild-moderate or severe pain (**A**) or disability (**B**) Number are n (%) and n _Total_=570

Figure 2 shows the Sankey diagram of the 4-year courses of pain (2A) and disability (2B). Of the 241 participants included in the analysis of the Sankey diagrams 130 (54%) had mild-moderate pain, and 115 (48%) had mild-moderate disability consistently at all three time points, whereas only 17 (7%) had severe pain, and 37 (15%) had severe disability consistently at all three time points. Inspecting the Sankey diagrams, the patients with mild-moderate symptoms at baseline had an overall more stable course between baseline and the 1- and 4-year follow-ups than those with severe symptoms at baseline.

### Prognostic factors for improvement in pain and disability

A total of 16 variables were included in the prognostic analyses. See Table 2 for details of the definitions and coding used in the analysis.

**Table 2:** Candidate prognostic factors for participants included in the study*

Variable	Question	Response option	Coding in analysis	
Age	Calculated from CPR and date of first visit	Years	Continuous	
Sex	Calculated from CPR	Female/Male	Categorical	
Normal weight [34]	1. Weight 2. Height	1. kg 2. cm	Categorical Calculated from weight and height. ‘Not normal weight’: BMI < 18.5 or > 24.9 < kg/m2;‘Normal weight’: BMI 18.5–24.9 kg/m2	
LBP intensity [23]	LBP now. Worst LBP intensity in the last 14 days. Typical LBP intensity in the last 14 days.	Numerical Rating Scale (NRS) (0–10) 0: No pain; 10: Worst possible pain	Continuous (0–10) Averaged on present LBP, worst LBP last 14 days and typical LPB last 14 days	
Episode duration	Approximately when was the onset of your low back pain or leg pain (sciatica)?	Days since onset of LBP	Continuous Months calculated from days since onset of LBP	
Previous LBP episodes	Have you had previous episodes of low back pain or leg pain (sciatica)?	Yes/No	Dichotomous	
Leg pain	Leg pain intensity now. Worst leg pain (sciatica) intensity in the last 14 days. Typical leg pain (sciatica) intensity in the last 14 days.	Numerical Rating Scale (NRS) (0–10) 0: No pain; 10: Worst possible pain	Dichotomous Presence of leg pain: > 0 on ≥ 1 item	
Pain in other parts	Over the last 2 weeks, have you been bothered by pain in body parts other than you have marked on the previous drawing?	Yes/No	Dichotomous	
Disability [35, 36]	23-item Roland Morris Disability Questionnaire (RMDQ)	Yes/No for each item	Converted to a proportional score (0-100) 0: No disability; 100: Extremely severe disability	
Depression [37–39]	During the past month have you often been bothered by feeling down, depressed, or hopeless? During the past month have you often been bothered by little interest or pleasure of doing things?	Ordinal (0–10)	Dichotomized: Not Depressed < 6; Depressed ≥ 6	
Anxiety [37]	Do you feel anxious?	Ordinal (0–10)	Dichotomized: Not anxious < 5; Anxious ≥ 5	
Fear-avoidance [37]	Physical activity might harm my back. I should not do physical activities which (might) make my pain worse.	Ordinal data (0–10)	Dichotomized: No fear-avoidance < 7; Fear-avoidance ≥ 7	
Risk of persistent pain [40]	In your view, how large is the risk that your current pain may become persistent?	Ordinal data (0–10) Dichotomized: High risk ≥ 7; Low risk < 7.	Categorical	
Social isolation [37]	Do you feel socially isolated?	Ordinal data (0–10)	Dichotomized: Not socially isolated < 2; Socially isolated ≥ 2	
Present work participation	What is your present work participation?	1: Regular employment (fulltime or part time); 2: Flex job; 3: Studying; 4: Undertaking rehabilitation; 5: Unemployed; 6: Receiving disability pension; 7: Receiving retirement pension; 8: Housewife or househusband; 9: Other	Dichotomized: ‘No regular employment or studying’ 2, 4–9; ’Regular employment or studying’ 1 or 3	
Sick leave	Have you taken sick leave for low back pain or leg pain (sciatica) in the last 3 months?	Yes/No	Dichotomous	

*Lower scores are better on all questionnaire scales.

BMI: Body Mass Index, RMDQ: Roland Morris Disability Questionnaire (23-item version); NRS: Numerical Rating Scale.

Of the 570 participants with data on pain and disability at baseline and the 4-year follow-up, 46 were excluded due to their inability to improve (baseline NRS < 2 and RMDQ < 5), leaving 524 participants for inclusion in the logistic regression model. Improvements in pain and disability were observed in 44% (95% CI; 40–48) and 48% (44–53) of the included participants, respectively. There was an overlapping group of 32% (28–36) who improved in both pain and disability.

The results of the univariate logistic regression on baseline prognostic factors for improvement in pain and disability at 4 years are shown in Table 3. None of the 16 variables had > 10% missing values and were therefore all included in the univariate analysis. Eight variables (age, sex, pain intensity, disability, previous episodes of LBP, episode duration, risk of persistent pain, and regular employment or studying) met the criteria of a *p*-value ≤ 0.2 for further inclusion in the multivariate analysis. The correlation coefficients of all variables assessed were less than 0.5 and therefore collinearity was not considered a concern.

**Table 3:** Univariate analyses of baseline prognostic factors for improvement in pain and disability at 4 years

Candidate prognostic factors	OR (95% CI)	*P*-value
Age (years)	**0.97 (0.94-1.00)**	**0.087**
Sex (female)	**1.31 (0.90–1.90)**	**0.156**
Normal weight (BMI)	0.93 (0.64–1.36)	0.708
Pain intensity (NRS)	**1.26 (1.12–1.42)**	**< 0.0001**
Episode duration (months)	**0.99 (0.98-1.00)**	**0.001**
Previous LBP episodes	**0.73 (0.47–1.13)**	**0.154**
Leg pain	0.88 (0.54–1.45)	0.625
Pain in other parts	0.80 (0.55–1.16)	0.243
Disability (RMDQ)	**1.02 (1.01–1.02)**	**0.001**
Depression	1.12 (0.72–1.72)	0.617
Anxiety	1.19 (0.82–1.71)	0.360
Fear-avoidance	0.95 (0.61–1.50)	0.838
Risk of persistent pain	**0.63 (0.44–0.92)**	**0.016**
Social isolation	1.00 (0.66–1.52)	0.983
Regular employment or studying	**1.43 (0.95–2.16)**	**0.089**
Sick leave	0.95 (0.65–1.40)	0.813

n _Total_=570 (varies due to missing values)

OR: Odds ratio, BMI: Body Mass Index, RMDQ: Roland Morris Disability Questionnaire (23-item version), NRS: Numerical Rating Scale

Bold font indicate *p*-value ≤ 0.2

The results of backward stepwise multivariate logistic regression (model 1) are shown in Table 4. Five variables were significantly associated with improvement in pain and disability at 4 years; positive associations were observed for pain intensity (OR 1.34 [95% CI;1.17–1.54]), disability (OR 1.01 [1.00-1.02]), and regular employment or studying (OR 1.67 [1.06–2.64]), and negative associations with the outcome were observed for both episode duration (OR 0.99 [0.99-1.00]) and risk of persistent pain (OR 0.58 [0.38–0.88]).

The Hosmer-Lemeshow goodness of fit test yielded a *p* > 0.05, suggesting an acceptable model fit.

**Table 4:** Multivariate analyses of baseline prognostic factors for improvement in pain and disability at 4 years (model 1)

Prognostic factors	OR (95% CI)	*P*-value
Age (years)	–	–
Sex (female)	–	–
Pain intensity (NRS)	1.34 (1.17–1.54)	< 0.0001
Episode duration (months)	0.99 (0.99-1.00)	0.009
Previous LBP episodes	–	–
Disability (RMDQ)	1.01 (1.00-1.02)	0.015
Risk of persistent pain	0.58 (0.38–0.88)	0.011
Regular employment or studying	1.67 (1.06–2.64)	0.027

n _Total_=498 (72 participants were excluded due to missing values in the include variables).

OR: Odds ratio, BMI: Body Mass Index, RMDQ: Roland Morris Disability Questionnaire (23-item version), NRS: Numerical Rating Scale.

In the multivariate non-stepwise regression including all 16 variables (Additional file 2; model 2) and the extended stepwise regression including all variables (Additional file 2; model 3) the same five prognostic factors as in model 1 were found statistically significant. The AUCs for all three multivariate regressions ranged from 0.70 to 0.71, and the test for equality of the ROC curves yielded a *p* = 0.214. Hence, there was no significant difference in the discriminatory ability of the 3 regression models.

## Discussion

In the current study of patients with LBP in a secondary sector care setting, approximately half of the participants had consistent mild-moderate levels of pain and disability at baseline, 1-year, and 4-year follow-up. Conversely, only a small percentage had severe pain and disability at all three measurement points, suggesting that only a small percentage of this LBP population had persistent severe symptoms. Furthermore, the patients with mild-moderate symptoms at baseline had a more stable course overall than those with severe symptoms at baseline. Regarding symptom improvement, we found that in this study population, approximately half of the patients improved over 4-years, with similar proportions for pain and disability. Finally, we identified five baseline prognostic factors for improvement in pain and disability at the 4-year follow-up: pain intensity, disability score, episode duration, risk of persistent pain, and regular employment or studying.

Overall, our results on the progression of pain and disability are consistent with previous studies showing that LBP patients do not necessarily have the same symptom levels throughout the follow-up period [9, 10]. In the current study, about half of the patients (54% and 48%, respectively) had mild-moderate levels of pain and disability consistently at all three time points, in line with previous studies in both primary care [8–10] and general populations [19, 41, 42]. Furthermore, our finding that patients can improve in pain, disability, and both is consistent with previous studies [8–10, 19, 41, 42] and highlights the potential for positive long-term outcomes in this population. These results add to the understanding of the heterogeneous course of persistent LBP, with subgroups having varying symptom severity and progression. Future long-term studies in the secondary care setting, using repeated follow-up measurements and analysing trajectories using time series and clustering techniques, may provide a better understanding of the heterogeneity and variability of LBP over time [4].

Finally, we found five prognostic factors for improvement in pain and disability. One of these factors was reporting of a perceived risk of persistent pain, which was associated with lower odds of improvement at 4years (OR 0.58), reflecting a 42% decreased chance of improvement for those at higher risk. Different definitions of expectations and perceptions have also previously been associated with LBP outcomes in primary care studies [8, 18, 43–45]. This may be explained by the influence of perceptions on subsequent responses and behaviours that affect prognosis. Pain beliefs are one component in the biopsychosocial model that addresses the complexity of LBP, and unaddressed baseline factors influencing these beliefs may have become current issues over time [46–48].

Notably, depression and fear-avoidance were not significant prognostic factors in our study, although they have been reported to be relevant in several previous studies investigating LBP outcomes [9, 49, 50]. The differing results between our study and previous studies may be due to differing follow-up periods and study populations. Psychological characteristics have been proposed to contribute to LBP at different time points, and suggestively depression is more predictive of the 12-month outcome in patients with acute LBP compared to chronic LBP [51].

Moreover, regular employment or studying significantly predicted a 4-year improvement in the current study (OR 1.67). In other words, the likelihood of experiencing improvement in pain and function is 67% higher for the patients categorized as having regular employment or studying compared to those who are not. Similar results have been reported in two primary care studies, including patients with mixed LBP duration, where unemployed participants had less favourable 12-month outcomes [14, 15]. The lower odds of improvement in patients not having regular employment or studying may be related to unstudied circumstances, such as unemployment due to severe LBP [52].

Episode duration (OR 0.99) also predicted improvement in our study, reflecting a 1% lower chance of improvement for each additional month of episode duration. This finding aligns with a previous long-term primary care study [10], strengthening the significance of episode duration as a prognostic factor. In this earlier study, episode duration was associated with higher Oswestry Disability Index scores at both 1 and 5 years (OR 2.36 and OR 1.79, respectively) [10]. These findings imply that the duration of episodes of LBP is not just a short-term concern; individuals with longer episodes of LBP tend to face greater challenges in achieving significant improvements in their condition.

As expected, baseline pain intensity (OR 1.34) and disability (OR 1.01) predicted improvement in pain and disability at 4 years, in line with previous studies [8, 9, 13]. In the current study, each unit increase in baseline pain intensity and disability increased the odds of improvement in symptoms by 34% and 1%, respectively. The associations between higher baseline outcome-related variables and greater odds of improvement may be because starting from a higher baseline score will allow for a more substantial reduction, a pattern also observed in previous studies [13, 53].

This study represents an initial step in prognostic research aiming to identify factors associated with long-term improvement in patients with persistent LBP. Identification of modifiable and non-modifiable prognostic factors is essential to determine potential targets for the treatment of LBP [6, 7, 29]. In the current study, we identified prognostic factors for future consideration, and confirmatory studies are needed to investigate causal determinants and develop prognostic models that predict long-term improvement in patients with persistent LBP. This would help inform patients about their future course of LBP and guide clinicians in making decisions about further treatment [6, 7, 29].

The primary strength of this study is its 4-year follow-up period, as long-term prognostic studies in the LBP population are limited. However, a long follow-up period with measurements at baseline and 4 years only also presents certain limitations, such as the risk of misclassification [54]. Patients with LBP tend to follow different trajectories, and their symptom severity may fluctuate over time [4, 5]. Hence, an improvement or a worsening of symptoms observed at the time of measurement may reflect a short episode of improvement or worsening rather than the complete picture. Thus, measuring the outcome at multiple time points would likely have provided a more accurate estimate of improvement, but this was not feasible with the current pre-established dataset. Other methodological strengths include the use of validated outcome measures and commonly used cutoff points. However, there are inconsistencies in the definition of cutpoints across studies, which may affect generalisability, including the current study.

A limitation is the relatively high rate of participants lost to follow-up, which may have affected the overall reliability and generalisability of the findings. Having fewer participants at follow-up compared to baseline may have introduced a bias in representativeness if the participants lost to follow-up differed from those retained. Additionally, this reduction in sample size may result in less precise information about the outcomes over time and could lead to a loss of statistical power, affecting the ability of the study to detect changes or associations. Another limitation is that potentially relevant prognostic factors were not included. Consequently, we cannot draw conclusions about the effect of variables not included in our analysis. A further limitation is the dichotomisation of several numerical prognostic variables. Dichotomization can lead to extreme rounding and loss of valuable information and power [55], but it facilitated the interpretation of our results. Dichotomization can lead to extreme rounding and loss of valuable information and power [55], but it facilitated the interpretation of our results.

## Conclusion

This study found that patients with persistent LBP in secondary care can improve in pain and disability over 4 years. While only a small proportion had consistently severe pain and disability at all three time points, a large group had consistently mild-moderate pain and disability. Moreover, we identified five prognostic factors of long-term improvement in LBP: pain intensity, disability, episode duration, regular employment or studying, and risk of persistent pain. To strengthen the reliability and significance of these findings, additional confirmatory and causation studies are needed. Such studies would validate the observed prognostic factors and their relevance to long-term improvement in LBP. By building on these findings through further research, we can gain a deeper understanding of the factors that influence the course of LBP and potentially improve treatment strategies to optimize patient outcomes.

### Electronic supplementary material

Below is the link to the electronic supplementary material.

Supplementary Material 1

## Data Availability

The datasets used/or analysed during the current study are available from the corresponding author on reasonable request.

## Abbreviations

LBP: Low back pain
SSD: Spines of Southern Denmark
NRS: Numeric Rating Scale
RMDQ: Roland Morris Disability Questionnaire
AUC: Area under the curve
IQR: Interquartile range
95% CI: 95% confidence interval
OR: Odds ratio
