# Supplementary material for: Prognostic factors for long-term improvement in pain and disability among patients with persistent low back pain
Source: Chiropr Man Therap. 2024 Jun 25;32:26. doi: 10.1186/s12998-024-00546-z (PMC11202371; doi:10.1186/s12998-024-00546-z)
Supplement: Supplementary file 1 — Supplementary Material 1 [file 12998_2024_546_MOESM1_ESM.docx]

## ADDITIONAL FILES

## Additional file 1

## Baseline characteristics of the included and not-included in the 1- and 4-year follow-up study.

| **Baseline variables ^a^** | **Included** | | **Not included ^b^** | |
| --- | --- | --- | --- | --- |
|  | **Missing values,**  **n (%)** |  | **Missing values,**  **n (%)** |  |
| *Contextual factors* |  |  |  |  |
| Age (years), median (IQR) | 0 (0) | 33.9 (29.0-37.7)* | 0 (0) | 32.2 (26.2-36.4)* |
| Sex (female) | 0 (0) | 51 (45.1-57.8) | 0 (0) | 55 (51.1-58.0) |
| Normal weight (BMI) | 6 (2.5) | 45 (38.6-51.5) | 105 (13.2) | 45 (41.7-49.2) |
| *Pain-related factors* | | | | |
| Pain intensity (NRS), median (IQR) | 0 (0) | 6 (4.7-7.3) | 59 (7.4) | 6.3 (5.0-7.3) |
| Episode duration (months), median (IQR) | 7 (2.9) | 8.6 (3.3-32.0) | 75 (9.4) | 11.1 (4.0-39.0) |
| Previous episodes | 3 (1.2) | 82 (76.5-86.5)* | 68 (14.6) | 72 (68.7-75.2)* |
| Leg pain | 2 (0.8) | 83 (78.5-88.0) | 71 (8.9) | 80 (76.9-82.8) |
| Pain in other parts | 4 (1.7) | 44 (37.9-50.7) | 77 (9.7) | 40 (36.2-43.4) |
| *Activity limitation restricted factors* | | | | |
| Disability (RMDQ), median (IQR) | 0 (0) | 52.17 (39.1-73.9) | 76 (9.5) | 61 (43.5-73.9) |
| *Psychological factors* | | | | |
| Depression | 4 (1.7) | 22 (16.6-27.2) | 91 (11.4) | 22 (19.2-25.3) |
| Anxiety | 3 (1.2) | 43 (36.5-49.2) | 88 (11.1) | 45 (41.7-49.0) |
| Fear-avoidance | 2 (0.8) | 17 (12.0-21.5)* | 80 (10.0) | 26 (22.4-28.8)* |
| Risk of persistent pain | 3 (1.2) | 46 (39.4-52.2) | 88 (11.1) | 46 (42.1-49.4) |
| Social isolation | 8 (1.4) | 12 (8.0-16.4) | 88 (11.1) | 13 (10.8-15.6) |
| *Participation restricted related factors* | | | | |
| Regular employment or studying | 3 (1.2) | 75 (69.3-80.5)* | 82 (10.3) | 67 (63.5-70.4)* |
| Sick leave | 16 (6.6) | 48 (41.4-54.6) | 127 (16.0) | 51 (47.2-54.8) |
| Included participants; n=241, Not-included participants; n=796.  a: Except where indicated otherwise, values are the percent of patients (95% confidence interval).  b: Participants included in the baseline cohort, but not in the analysis of the Sankey diagrams.  IQR: interquartile range, BMI: Body Mass Index, RMDQ: Roland Morris Disability Questionnaire (23-item version); NRS: Numerical Rating Scale.  *P-value <0.05 | | | | |

## Additional file 2

## Alternative logistic regression models (model 2 and 3)

## Multivariate analyses of baseline prognostic factors for improvement in pain and disability at 4 years (model 2).

| **Prognostic factors** | **OR (95% CI)** | **P-value** |
| --- | --- | --- |
| Age (years) | 0.98 (0.94-1.01) | 0.163 |
| Sex (female) | 1.03 (0.66-1.60) | 0.905 |
| Normal weight (BMI) | 1.03 (0.66-1.59) | 0.909 |
| Pain intensity (NRS) | 1.29 (1.11-1.50) | 0.001* |
| Episode duration (months) | 0.99 (0.99-1.00) | 0.017* |
| Previous LBP episodes | 0.97 (0.58-1.61) | 0.900 |
| Leg pain | 0.76 (0.42-1.36) | 0.354 |
| Pain in other parts | 0.82 (0.53-1.27) | 0.378 |
| Disability (RMDQ) | 1.02 (1.01-1.04) | 0.001* |
| Depression | 0.91 (0.51-1.62) | 0.758 |
| Anxiety | 1.05 (0.66-1.67) | 0.839 |
| Fear-avoidance | 0.80 (0.46-1.38) | 0.422 |
| Risk of persistent pain | 0.60 (0.38-0.96) | 0.033* |
| Social isolation | 0.93 (0.54-1.59) | 0.794 |
| Regular employment or studying | 1.68 (1.01-2.79) | 0.047* |
| Sick leave | 0.66 (0.41-1.04) | 0.070 |
| n _Total_=450 (120 participants were excluded due to missing values).  OR: Odds ratio, BMI: Body Mass Index, RMDQ: Roland Morris Disability Questionnaire (23-item version), NRS: Numerical Rating Scale. *P-value <0.05. | | |

## Multivariate analyses of baseline prognostic factors for improvement in pain and disability at 4 years (model 3).

| **Prognostic factors** | **OR (95% CI)** | **P-value** |
| --- | --- | --- |
| Age (years) | ̶ | ̶ |
| Sex (female) | ̶ | ̶ |
| Normal weight (BMI) | ̶ | ̶ |
| Pain intensity (NRS) | 1.31 (1.13-1.51) | <0.0001 |
| Episode duration (months) | 0.99 (0.99-1.00) | 0.013 |
| Previous LBP episodes | ̶ | ̶ |
| Leg pain | ̶ | ̶ |
| Pain in other parts | ̶ | ̶ |
| Disability (RMDQ) | 1.02 (1.00-1.03) | 0.008 |
| Depression | ̶ | ̶ |
| Anxiety | ̶ | ̶ |
| Fear-avoidance | ̶ | ̶ |
| Risk of persistent pain | 0.62 (0.40-0.96) | 0.032 |
| Social isolation | ̶ | ̶ |
| Regular employment or studying | 1.79 (1.09-2.91) | 0.020 |
| Sick leave | ̶ | ̶ |
| n _Total_=450 (120 participants were excluded due to missing values). OR: Odds ratio, BMI: Body Mass Index, RMDQ: Roland Morris Disability Questionnaire (23-item version), NRS: Numerical Rating Scale. | | |
